# Type I Gaucher disease with exophthalmos and pulmonary arteriovenous malformation

**DOI:** 10.1186/1471-2350-6-25

**Published:** 2005-06-09

**Authors:** Chun-An Chen, Nelson LS Tang, Yin-Hsiu Chien, Wei-Min Zhang, Jou-Kou Wang, Wuh-Liang Hwu

**Affiliations:** 1 Departments of Pediatrics, National Taiwan University Hospital and National Taiwan University College of Medicine, Taipei, Taiwan; 2 Medical Genetics, National Taiwan University Hospital and National Taiwan University College of Medicine, Taipei, Taiwan; 3 Department of Chemical Pathology, The Chinese University of Hong Kong, Hong Kong

## Abstract

**Background:**

Gaucher disease type I, the non-neuropathic type, usually presents in adulthood with hepatosplenomegaly. We report here an adult with type I Gaucher disease presented with unusual and severe clinical manifestations.

**Case presentation:**

Hepatosplenomegaly, bone crisis and fractures occurred at early childhood, and splenectomy was performed at the age of 5. Exophthalmos with increase in retrobulbar space was noted when the patient was 30. Cerezyme infusion started at the age of 32; but unfortunately, pulmonary arteriovenous malformation with dyspnea and hypoxemia was found two years later. Gene analysis revealed V375L/L444P mutations in the β-glucocerebrosidase gene.

**Conclusion:**

Although both eye and lung diseases have been associated with Gaucher disease, this is the first reported demonstration of exophthalmos and pulmonary arteriovenous malformation in the same patient. This case may therefore present an extremely severe and unusual form of type I Gaucher disease.

## Backgound

Gaucher disease is a lysosomal storage disorder caused by a recessively inherited deficiency of glucocerebrosidase activity, which causes an accumulation of sphingolipid glucosylceramide in cells of the reticulo-endothelial systems [1]. The "Gaucher cells" can be found in the spleen, liver, bone, and central nerve system in affected individuals, causing hepatosplenomegaly, anemia, thrombocytopenia and skeletal diseases [1]. Three clinical forms of the disease have been described, based on the absence (type I) or the presence (types II and III) of neurological involvement in addition to the visceral findings [1].

Pulmonary involvement, with Gaucher cell infiltration of the alveolar or interstitial spaces [2], may be more common than previously thought, but clinically significant lung disease is still rare [3]. Pulmonary arteriovenous shunting has been implicated as the etiology of hypoxemia in patients with long-standing liver disease related to Gaucher disease [4,5]. Pulmonary arteriovenous malformation (AVM) in Gaucher disease, however, has not been reported in the English literature. Ocular manifestations of type I Gaucher disease include infiltration of the retina, conjunctiva and uvea with visual loss [6-8]. However, exophthalmos related to Gaucher disease has also not been described. Here we report an adult with Gaucher disease complicated with these two unusual manifestations, exophthalmos and pulmonary AVM.

## Case presentation

This 34-year-old woman was a case of Gaucher disease type I with initial presentation of hepatosplenomegaly and severe bone diseases including fractures of the lower extremities and bone crisis at childhood. She was the second of a pair of twins. Her twin sister and another younger sister are both affected. There is no consanguinity in the family. She underwent splenectomy at age 5 years due to persistent thrombocytopenia, but the diagnosis of Gaucher disease was established only after a bone marrow examination at age 11 years. Her leukocytes β-glucocerebrosidase activity checked at age 26 years was 1.56 nmol/mg/h (normal: >28.42 nmol/mg/h). At that time, she had severe bone deformities, hepatomegaly, anemia, clubbing fingers and toes, but pink lips. There was no audible heart murmur, and her breathing sound was clear. Her liver size was measured 5 cm below subcostal margin at right mid-clavicular line, and neither spider angiomata nor superficial vein engorgement was found. Hemogram revealed a platelet count of 103 × 10^9^/L and hemoglobin level of 9.6 mg/dL. DNA analysis of this patient and her two affected siblings all revealed a V375L/L444P genotype of the β-glucocerebrosidase gene.

**Figure 1:**
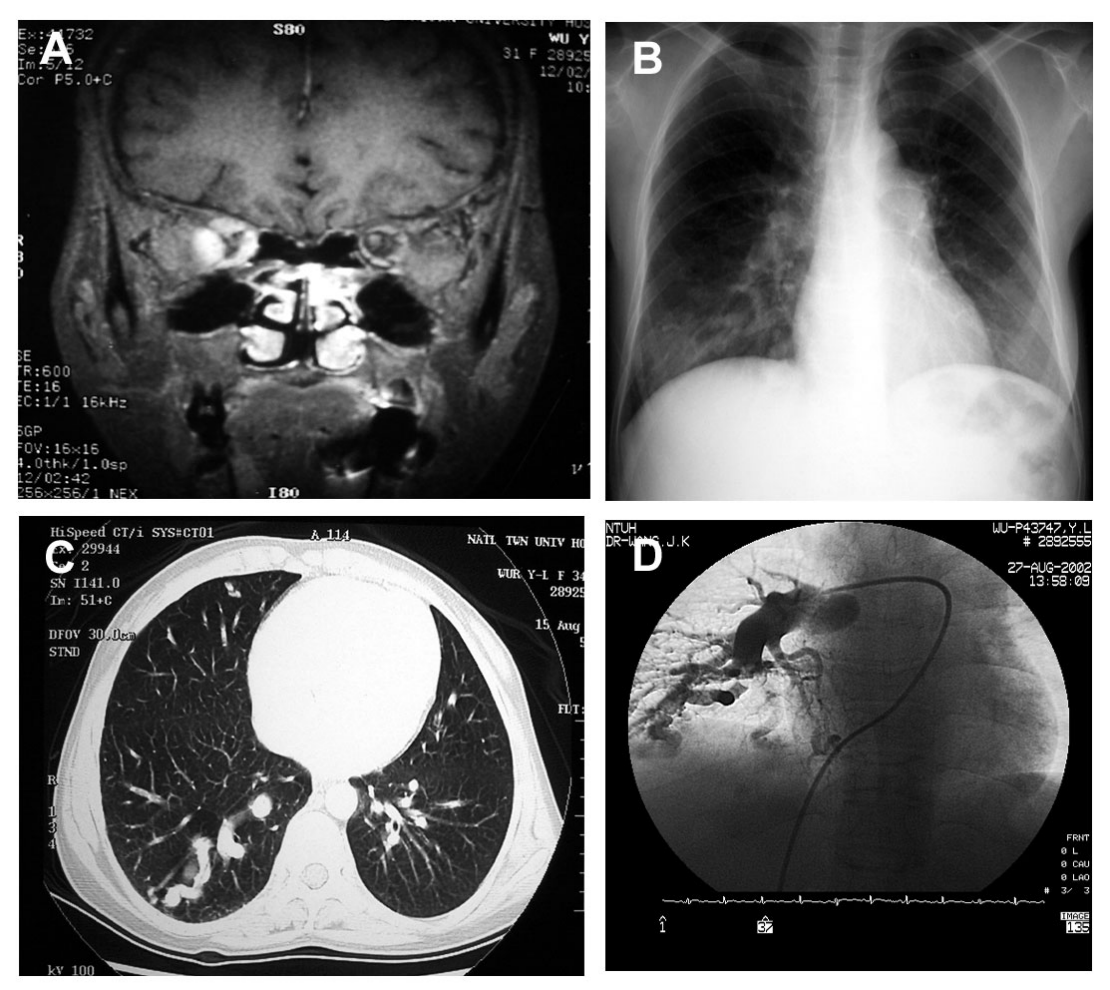
Type I Gaucher disease with exophthalmos and pulmonary arteriovenous malformation. (A) MRI of the head shows increases in retrobulbar space and hypertrophy of extra-ocular muscles. (B) Chest X ray reveals prominent pulmonary conus and increased infiltration over right lower lung field. (C) High-resolution computed tomography of chest reveals engorged right inferior pulmonary artery and its draining veins. (D) Pulmonary angiogram demonstrates right pulmonary arteriovenous malformation.

The patient complained of bilateral orbital pain with gradual protrusion of eyes since 30 years old. Thyroid function tests, including T3, T4, thyroid-stimulating hormone and free T4, showed normal results. Ophthalmologic examinations failed to reveal any specific findings related to the exophthalmos. Magnetic resonance imaging (MRI) of the eyes revealed increases in retrobulbar space with fat-like density and mild hypertrophy of extra-ocular muscles (Fig. 1A). She received enzyme replacement therapy (ERT) with imiglucerase (Cerezyme) at a dose of 60 U/kg every two weeks since 32 years old. Her exophthalmos progressed slightly during the first year of ERT, and then started to regress. Exposure conjunctivitis still bothered her currently.

The patient suffered from intermittent dyspnea at age 34 years. She became cyanotic and had an oxygen saturation of 85% as measured by pulse oximeter. Arterial blood gas analysis revealed a pH of 7.41, carbon dioxide tension of 27.9 mmHg, and oxygen tension of 61.2 mmHg in room air. A grade III/VI bruit was audible over the right lower chest, and a chest x-ray revealed prominent pulmonary conus and increased infiltration over the right lower lung field (Fig. 1B). A high-resolution computed tomography (HRCT) of the chest suspected an AVM with engorged right inferior pulmonary artery and its draining veins (Fig. 1C); but no focal lesions were found in the lung parenchyma. Right pulmonary artery angiogram showed several dilated and tortuous vessels from the right pulmonary artery, which directly connected to vessels draining into the right pulmonary vein over the right lower lobe of the lung (Fig. 1D). Early appearance of contrast medium in the right pulmonary vein indicated the presence of pulmonary arteriovenous malformation. The patient's pulmonary artery pressures were 48/19 mmHg (mean, 33 mmHg). Embolization was achieved with 6 coils, and resulted in a rise of oxygen saturation to 96%, although residual shunt was still present. Unfortunately, two months later, dyspnea and cyanosis recurred, and her oxygen saturation dropped to 85% in room air. A pulmonary function test at that time revealed moderately severe restrictive lung disease.

## Discussion

This 34-year-old patient presented symptoms and signs of exophthalmos and pulmonary AVM, which are unusual for Gaucher disease. She had normal mentality with no neurological symptoms such as ophthalmoplegia, therefore the disease could be classified as type I. However, she had severe thrombocytopenia requiring splenectomy and had had repeated fracture requiring prolonged immobilization. In a recent report from the Gaucher Registry, fracture was present in only 15% of all patients [9]. The patient's twin sister received splenectomy at young age and has bone disease similar to hers. Therefore, these sisters have an illness toward the severe end of the type I Gaucher disease.

Ocular manifestations in Gaucher disease are very rare, and the tissues reported to be involved are retina, conjunctive, and uvea [6-8]. In the MRI study, the increased retrobulbar spaces are filled with tissues with fat density, suggesting that the exophthalmos is caused by Gaucher cell infiltration. The clinical course also supports this hypothesis, since the patient's eyes started to retract after ERT.

Gaucher cells can infiltrate the alveolar spaces or the interlobular and intralobular septa, leading to air space and interstitial disease [2]. Gaucher cells can also plug in the pulmonary capillary vessels and cause pulmonary hypertension [10]. However, the chest x-ray and HRCT of the index patient revealed normal lung parenchyma, not suggesting direct alveolar or interstitial infiltration of Gaucher cells. It is also known that Gaucher disease can cause hepatic dysfunction which may induce abnormal dilatation of the intrapulmonary capillaries or the so-called hepatopulmonary syndrome [4,5]. It is possible that pulmonary AVM was the consequence of an abnormal progression of the hepatopulmonary syndrome, however, there was no overt evidence of severe hepatic dysfunction in the patient.

ERT has had a great impact on the outcome of Gaucher disease [11]. On the other hand, the effect of ERT on pulmonary hypertension remains to be established [12-15]. The patient had mild pulmonary hypertension. The pulmonary hypertension might not be related to AVM, since the latter condition usually causes low pulmonary artery pressure [16]. It has been reported that pulmonary hypertension may be triggered or aggravated by ERT [17]. It is possible that the pulmonary AVM may have existed but only caused symptoms when ERT changed intrapulmonary hemodynamics after clearance of Gaucher cells.

Through a registry of 1698 patients reported in 2000, the allele frequency of N370S was 53%, and that of L444P was 18% [9]. The L444P mutation is more common in Asians [19,20], and has been detected in Taiwanese patients with both type I and II Gaucher disease [20,21]. The prevalence of L444P and the absence of the N370S mutation may explain the more severe phenotype in Gaucher disease in Asians. The V375L mutation has been classified as a mild mutation [22], which might explain the V375L/L444P genotype in type I Gaucher disease. However, although the twin sister of the indexed person had bone disease of similar severity, she didn't have eye or lung problem. One the contrary, their younger sister has less skeletal involvemen, but had dyspnea and cyanosis, which responded to ERT. There surely are non-allelic or epigenetic factors influencing the phenotypes.

## Conclusion

Although both eye and lung diseases have been associated with Gaucher disease, this is the first reported demonstration of exophthalmos and pulmonary AVM in the same patient. This case may therefore present an extremely severe and unusual form of type I Gaucher disease. Different responses of these lesions to ERT would probably be attributed to different pathogenesis and natural course in the organ involvement in Gaucher disease.

## Competing interests

The author(s) declare that they have no competing interests.

## Authors' contributions

CC prepared the manuscript of this case report. YC and WH conducted long-term follow-up and prescribed ERT for the patients. NT and WZ carried out the gene mutation analysis. JW performed coil embolization of the pulmonary AVM. All authors read and approved the final manuscript.

## Pre-publication history

The pre-publication history for this paper can be accessed here:
